# Mechanistic gaps and statistical considerations in pulmonary heart disease: Insights from PAH combination therapy studies

**DOI:** 10.1002/ehf2.15385

**Published:** 2025-07-17

**Authors:** Youtao Zhang

**Affiliations:** ^1^ Division of Geriatrics The First Affiliated Hospital of Hebei North University Zhangjiakou Hebei Province China

Dear Editor,

Skowasch et al. present an insightful analysis comparing mono and combination therapies in patients with pulmonary arterial hypertension (PAH), as documented in their recent study.^1^ Their work offers significant contributions to optimizing therapeutic strategies in PAH, a field characterized by complex treatment paradigms and evolving evidence bases. Nonetheless, there are several aspects within the study that warrant further discussion.

First, the COMPERA studies have identified short‐term benefits and long‐term failures in PAH treatments, yet they have not sufficiently elucidated the underlying mechanisms. Specifically, the study only focused on downstream factor changes such as BNP/NT‐proBNP and ignored upstream pathways (e.g., endothelin or inflammatory signalling),^2^ limiting clinical translation. To address this gap, I suggest that the authors consider conducting organoid experiments to mimic PAH and co‐morbid environments. Specifically, patient‐derived pulmonary arterial organoids could be used to investigate how dual endothelin‐receptor blockade affects SMAD‐dependent signalling, providing a clearer link between the clinical results and the biological mechanisms.^3^ Transcriptomics combined with CRISPR editing can further identify key pathways (e.g., TGF‐β). Specifically, metabolomics studies could be employed to identify specific metabolic markers, such as elevated levels of long‐chain acylcarnitines like palmitoylcarnitine (C16) and stearoylcarnitine (C18), which have been associated with adverse outcomes in PAH.^4^ Additionally, host–microbe interaction studies could explore whether specific alterations in the gut microbiome, such as increased abundance of pro‐inflammatory bacterial taxa, are linked to treatment response heterogeneity in PAH patients.

The statistical analysis of this study raises concerns that multiple hypothesis testing may lead to higher type I error rates. The original article was scrutinized in order to determine whether Skowasch et al. presupposed endpoints. The authors noted that their analysis was exploratory and did not presuppose an endpoint. Given this exploratory nature, it is important to recognize the possibility of inflated type I errors when interpreting the results.

The study tested four primary endpoints (WHO functional class, 6‐min walk distance, NT‐proBNP level and risk profile). In the study, 43 (24.9%) patients in the monotherapy group and 67 (37.0%) patients in the combination therapy group showed improvement in WHO functional class, with *P*‐value of 0.0299. Additionally, the relative change in NT‐proBNP/BNP from baseline to first follow‐up was −28.3% [−72.3%, 66.9%] for monotherapy and −57.0% [−83.9%, −6.8%] for combination therapy, with a *P*‐value of <0.0001. The change in risk status showed that 71 (39.2%) patients in the monotherapy group and 100 (52.6%) patients in the combination therapy group had improvement, with a *P*‐value of 0.0285. Multiplicity adjustment may not always be mandatory in such exploratory studies, but discussing other methods, such as stratified tests, gatekeeping strategies or combined models with repeated measures, would strengthen the analysis. For example, stratified tests can prioritize testing of primary endpoints and test secondary endpoints only if the primary outcome is significant, thereby controlling the overall type I error rate more effectively.

The study by Skowasch et al. provides useful findings for PAH combination therapy. However, more work is needed to explore mechanisms and refine statistical methods. Future studies integrating multi‐omics and experimental validation could improve our understanding of the causal mechanisms and improve precision medicine for patients with PAH.

## Funding

Not applicable.

## Data Availability

Not applicable.
